# Artificial intelligence-driven therapeutics for disease modification in type 1 diabetes: a digital public health and clinical translation framework

**DOI:** 10.3389/fpubh.2026.1915502

**Published:** 2026-09-24

**Authors:** Krish Hirani, Camila Blaschke, Carlota Olloqui, Rahul Mittal

**Affiliations:** 1 Diabetes Research Institute, University of Miami Miller School of Medicine, Miami, FL, United States; 2 Division of Endocrinology, Diabetes, and Metabolism, Department of Medicine, University of Miami Miller School of Medicine, Miami, FL, United States

**Keywords:** artificial intelligence, digital public health, disease modification, immune tolerance, population health, precision medicine, type 1 diabetes, β-cell preservation

## Abstract

Type 1 diabetes (T1D) is a chronic autoimmune disorder characterized by immune-mediated destruction of pancreatic β-cells, resulting in absolute insulin deficiency and lifelong dependence on exogenous insulin therapy. As a chronic condition with substantial population-level clinical, psychosocial, and economic consequences, T1D represents an important public health challenge that extends beyond individual glycemic management. Although major advances in insulin formulations, continuous glucose monitoring systems, and automated insulin delivery technologies have substantially improved glycemic control and reduced complications, these approaches remain fundamentally disease-management strategies and do not address the underlying autoimmune pathology or restore durable endogenous insulin secretion. Over the past several decades, multiple clinical trials targeting immune modulation, β-cell preservation, transplantation, and metabolic pathways have generated significant mechanistic insights but have rarely achieved sustained insulin independence or long-term disease remission. Progress toward disease-modifying and prevention-oriented therapies has been hindered by biological heterogeneity, limited mechanistic biomarkers, translational gaps between experimental models and human disease, and fragmented data ecosystems. Advances in artificial intelligence (AI) and machine learning (ML) enable integrative analysis of multi-omics, clinical, and digital health datasets to define disease endotypes, predict disease trajectories, and guide the rational design of combination therapies and adaptive clinical trials. Within a digital public health framework, these approaches may also support earlier risk stratification, improved therapeutic targeting, more efficient trial design, and scalable implementation across diverse populations. However, the clinical translation of AI in T1D remains constrained by heterogeneity and fragmentation across available datasets, limited external validation, and inadequate representation of demographically and clinically diverse populations. Additional barriers include data privacy and governance requirements, the risk of algorithmic bias, and the need for interpretable and reproducible AI models that can meet rigorous clinical, ethical, and regulatory standards. This review synthesizes current knowledge of T1D pathophysiology and critically examines historical limitations in therapeutic development while proposing an AI-enabled precision medicine framework that integrates immune tolerance induction, β-cell restoration strategies, and metabolic optimization. Finally, we outline a translational roadmap for data-driven therapeutic discovery and adaptive clinical development aimed at accelerating progress toward durable, scalable, equitable, and biologically informed interventions for T1D with relevance to public health practice, prevention, and population health outcomes.

## 1 Introduction

Type 1 diabetes (T1D) is an immune-mediated metabolic disease defined by progressive autoimmune destruction of pancreatic β-cells, resulting in the loss of endogenous insulin secretion and chronic dependence on exogenous insulin therapy (1–5). The disease affects millions of individuals worldwide and represents a substantial global health burden associated with significant morbidity, premature mortality, and escalating healthcare costs (6–10). At the population level, these clinical consequences reflect not only the biological severity of β-cell failure but also the cumulative effects of delayed risk identification, variable access to advanced diabetes technologies, and long-term complication burden. Although modern insulin therapy has transformed survival outcomes since the discovery of insulin in the early 20th century, T1D remains a lifelong condition without a broadly available disease-modifying or curative therapy (11). Even with intensive glycemic management, individuals living with T1D continue to face elevated risks of long-term microvascular and macrovascular complications, including diabetic retinopathy, nephropathy, hearing loss, neuropathy, and cardiovascular disease (12–17). In addition to these medical risks, the daily management of T1D imposes considerable psychosocial and cognitive demands, requiring constant attention to glucose monitoring, insulin administration, dietary intake, physical activity, and the prevention of both hypoglycemia and hyperglycemia (18–22). Thus, T1D remains a major translational and public health challenge as current standards of care improve metabolic control but do not eliminate autoimmune activity, restore durable β-cell function, or fully prevent downstream complications across diverse patient populations.

Technological innovations over the past several decades have substantially improved the management of T1D. The development of recombinant insulin analogs, insulin pump therapy, continuous glucose monitoring systems, and hybrid closed-loop automated insulin delivery has enabled more precise glycemic regulation and has reduced the frequency of severe hypoglycemic events. These advances have brought clinical care closer to replicating physiological insulin delivery and have improved quality of life for many individuals with T1D. Nevertheless, these approaches remain fundamentally compensatory rather than curative. They address the metabolic consequences of β-cell loss but do not modify the underlying autoimmune processes responsible for disease initiation and progression. As a result, endogenous insulin production is rarely restored, and the lifelong therapeutic burden of disease management persists. This therapeutic gap highlights the need for strategies that move beyond glucose replacement toward immune modulation, β-cell preservation, β-cell restoration, and biologically informed prevention of disease progression.

The continued absence of a durable cure reflects the biological complexity of T1D pathogenesis. T1D arises from a multifactorial interplay between genetic susceptibility, dysregulated immune responses, intrinsic β-cell stress pathways, environmental exposures, and metabolic factors (23–27). Genetic susceptibility, particularly HLA and non-HLA variants, influences antigen presentation, immune regulation, and β-cell vulnerability (28–34). Environmental triggers, including viral infections, alterations in the intestinal microbiome, and early life exposures, may further shape immune tolerance and precipitate disease onset in genetically susceptible individuals (35–40). β-cells also actively contribute to disease progression through stress-induced signaling and altered antigen presentation that amplify immune recognition (23, 41). Accordingly, T1D is increasingly recognized as a heterogeneous disease process involving reciprocal interactions between immune dysregulation, β-cell intrinsic vulnerability, environmental perturbations, and metabolic stress rather than a uniform autoimmune phenotype.

Historically, therapeutic development in T1D has largely focused on targeting discrete components of the immune system, including T-cell activation, B-cell mediated immunity, and pro- inflammatory cytokine signaling. While several immunomodulatory therapies have demonstrated partial preservation of residual β-cell function, particularly when administered during early stages of disease, most interventions have produced only modest and transient clinical benefits. These outcomes highlight the limitations of reductionist therapeutic strategies in a disease governed by complex and highly interconnected immunological and metabolic networks. They also suggest that durable therapeutic benefit may require improved patient stratification, timing of intervention, mechanistic biomarker development, and rational combination approaches that account for disease stage and biological heterogeneity.

Recent advances in artificial intelligence (AI) and machine learning (ML) offer an unprecedented opportunity to address these challenges (42–44). Modern computational approaches enable the integration and analysis of large-scale heterogeneous datasets spanning genomics, transcriptomics, proteomics, metabolomics, immune phenotyping, longitudinal clinical data, and digital health signals generated by wearable devices and glucose monitoring technologies (43, 45). By extracting latent structure from these high dimensional datasets, AI methods can reveal previously unrecognized biological patterns, identify mechanistically distinct disease endotypes, and generate predictive models of disease progression and therapeutic response (46). Such approaches have the potential to transform T1D research by enabling precision stratification of patient populations, rational design of combination therapies, and more efficient clinical trial strategies. When embedded within translational and digital public health frameworks, AI-enabled systems biology may also support earlier identification of at-risk individuals, adaptive monitoring of disease trajectories, and scalable implementation of precision interventions in clinically diverse populations.

In this review, we integrate current understanding of the immunobiology and clinical heterogeneity of T1D and examine the major limitations that have historically constrained therapeutic progress. We then discuss how AI-enabled systems biology approaches can redefine disease classification, accelerate biomarker discovery, and inform the development of integrated therapeutic strategies aimed at restoring immune tolerance, preserving or replacing β-cell function, and stabilizing metabolic homeostasis (Figure 1). We outline emerging computational and clinical frameworks that could support adaptive, data-driven therapeutic development. These frameworks may accelerate progress toward durable and scalable disease-modifying interventions for T1D, with relevance to precision prevention, clinical translation, and population-level implementation.

**Figure 1:**
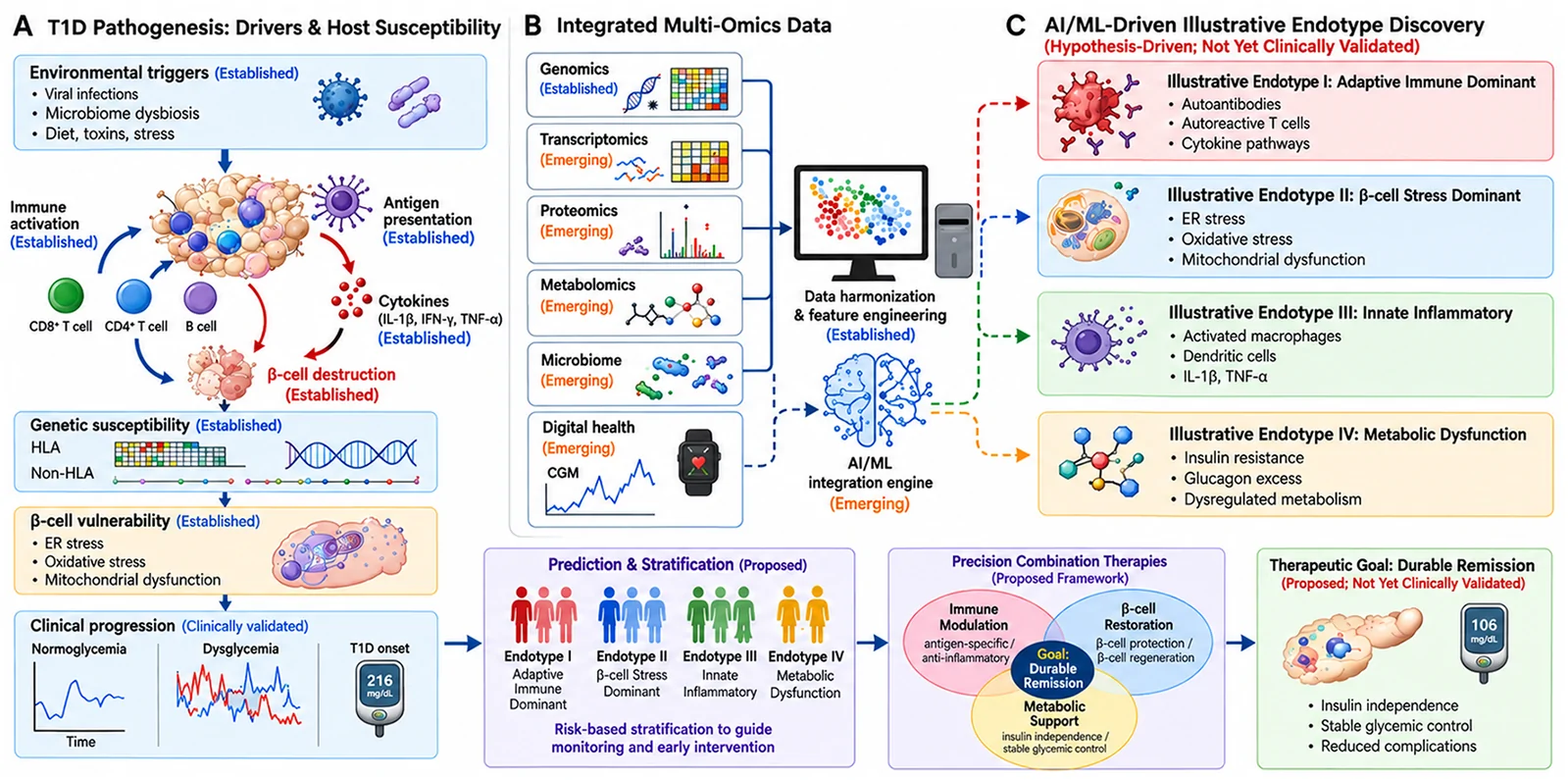
Systems biology framework linking disease pathogenesis, multi-omics integration, and AI-driven endotype discovery in type 1 diabetes (T1D). **(A)** Type 1 diabetes results from interactions between genetic susceptibility and environmental triggers that initiate immune activation and β-cell stress, leading to progressive autoimmune β-cell destruction; **(B)** Multi-omics data, including genomic, transcriptomic, proteomic, metabolomic, microbiome, and digital health datasets, are integrated using artificial intelligence and machine learning to characterize high-dimensional disease structure and infer candidate regulatory networks; **(C)** AI/ML-driven analyses may facilitate the discovery of proposed, hypothesis-driven disease endotypes, exemplified here by adaptive immune-dominant, β-cell stress-dominant, innate inflammatory, and metabolic dysfunction patterns. These proposed endotypes are conceptual and are not yet clinically validated or established as reproducible T1D subgroups. Such frameworks may ultimately support biological stratification and mechanism-informed therapeutic development. Created with Biorender.com.

### 1.1 Literature search strategy

This narrative review was informed by a structured literature search covering T1D pathophysiology, AI, ML, disease-modifying therapies, immunotherapy, β-cell preservation and replacement, multi-omics, digital health, precision medicine, and clinical trial design. Relevant peer-reviewed literature was identified using PubMed/MEDLINE, Scopus, Web of Science, and Google Scholar, with combinations of search terms including “type 1 diabetes,” “artificial intelligence,” “machine learning,” “deep learning,” “multi-omics,” “continuous glucose monitoring,” “precision medicine,” “immunotherapy,” “β-cell preservation,” “β-cell replacement,” “disease modification,” and “clinical trial.”

The literature represented in the final reference set spans 2014–2026, with emphasis on recent studies published during 2024–2026 to reflect current advances in AI-enabled disease characterization, therapeutic development, and clinical translation. The clinical and translational studies, randomized and interventional studies, prospective cohort studies, systematic reviews, meta-analyses, and relevant mechanistic investigations were prioritized according to their relevance to the scope of the review.

Additional publications were identified through examination of the reference lists of relevant articles and were included when they provided important mechanistic, methodological, or translational context. Studies were selected according to their relevance to T1D immunobiology and heterogeneity, AI- and ML-enabled disease characterization, multi-omics integration, biomarker discovery, therapeutic development, precision patient stratification, digital health, adaptive clinical trial design, and clinical translation.

As a narrative review, the literature selection was designed to provide a current and critical synthesis of evidence across interconnected biological, computational, and clinical domains. Particular emphasis was placed on studies that informed the proposed AI-enabled framework for disease endotyping, therapeutic discovery, precision trial design, and translation of disease-modifying strategies into clinically applicable approaches.

## 2 Historical challenges in developing disease-modifying therapies for T1D

Despite substantial advances in understanding the immunological and metabolic mechanisms associated with T1D, the development of therapies capable of producing durable disease remission has remained challenging. Over several decades, a wide range of experimental strategies have been investigated, including immunomodulatory agents, antigen-specific tolerance approaches, interventions designed to preserve pancreatic β-cell function, and emerging regenerative or cellular replacement therapies. While many of these interventions have contributed important insights into disease biology and have occasionally demonstrated partial preservation of endogenous insulin secretion, consistent long-term restoration of physiological insulin production has rarely been achieved. The gap between experimental progress and durable clinical outcomes reflects several factors that have historically complicated therapeutic development (Table 1). In particular, the biological processes underlying autoimmune β-cell destruction involve multiple interacting immune, cellular, and metabolic pathways, making it difficult for interventions directed at individual mechanisms to produce sustained effects. In addition, variability among individuals with T1D, limitations in available biomarkers for monitoring disease activity, and challenges associated with translating findings from experimental models into human clinical contexts have collectively influenced the pace of therapeutic progress. Recognizing these challenges provides useful context for interpreting past clinical studies and may help guide the design of future strategies aimed at achieving more durable modification of disease processes. From a public health perspective, these barriers are important because they constrain early identification of individuals most likely to benefit from intervention, limit efficient allocation of emerging therapies, and complicate equitable implementation of disease-modifying strategies across healthcare systems.

**Table 1:** Major barriers to therapeutic progress in type 1 diabetes.

Barrier	Biological/Clinical Cause	Consequence for trials	Potential AI-enabled solution	References
Monotherapy approaches	Networked immunometabolic disease	Transient benefits only	AI-guided combination therapy	(47, 58)
Disease heterogeneity	Genetic, immune, metabolic variability	Diluted treatment signals	Endotype stratification	(73, 123, 235, 241)
Biomarker limitations	Reliance on C-peptide and HbA1c	Delayed efficacy signals	Multi-omics biomarker integration	(82, 87, 112, 232)
Translational gaps	NOD mouse differences from humans	Failed translation	Humanized models + AI validation	(64, 67, 89, 90)

### 2.1 Monotherapy limitations in a networked disease

Many early therapeutic strategies for T1D were developed with the goal of modulating specific immune pathways thought to contribute to autoimmune destruction of pancreatic β-cells (47–49). These approaches were often based on the identification of individual molecular targets or immune cell populations involved in autoimmune activity, with the expectation that targeted intervention could disrupt disease progression (50–53). In some clinical investigations, therapies directed at particular immune mechanisms have demonstrated measurable biological effects, including temporary modulation of immune responses or partial preservation of residual β-cell function (54–56). However, the durability of these responses has generally been limited, and sustained remission has rarely been observed (57). One explanation for these outcomes is that autoimmune β-cell destruction appears to involve coordinated interactions among multiple immune and metabolic pathways rather than a single dominant mechanism (2, 58–60).

Current understanding suggests that T1D develops through complex interactions between adaptive immune responses, innate inflammatory signaling, and cellular stress processes within pancreatic β-cells (61–63). These components form interconnected biological networks in which changes within one pathway can influence several others (64–66). Consequently, interventions that target a single pathway may not fully disrupt the broader processes responsible for disease progression (67–69). In some cases, compensatory immune responses or alternative inflammatory signaling pathways may maintain autoimmune activity despite therapeutic intervention (70, 71). In addition, β-cells exposed to inflammatory or metabolic stress can exhibit changes in gene expression and cellular signaling that increase their susceptibility to immune-mediated injury (72). As these processes operate simultaneously, therapeutic strategies that focus exclusively on a single immune pathway may have limited ability to modify the overall disease environment in which β-cell destruction occurs (73). These observations have contributed to increasing interest in therapeutic approaches capable of addressing multiple biological mechanisms involved in disease progression. More broadly, they support therapeutic models that evaluate not only molecular efficacy but also intervention timing, patient selection, durability of response, monitoring requirements, and feasibility of implementation across clinically heterogeneous populations.

### 2.2 Disease heterogeneity

Another factor that has complicated therapeutic development is the considerable biological variability observed among individuals diagnosed with T1D (74–76). Although the disease is defined clinically by autoimmune destruction of pancreatic β-cells leading to insulin deficiency, the underlying biological processes involved in disease initiation and progression appear to vary among patients. Differences have been observed in genetic susceptibility patterns, immune regulatory pathways, metabolic physiology, and environmental influences that may contribute to disease development (36, 77, 78). As a result, individuals diagnosed with T1D may experience different rates of β-cell decline, variations in immune activation, and diverse responses to therapeutic intervention (79–81).

Variability is also observed in immune cell activity and pancreatic β-cell responses to stress. Differences in immune regulatory balance, cytokine signaling patterns, and β-cell resilience to inflammatory injury may influence both disease progression and therapeutic responsiveness. When individuals with diverse biological characteristics are enrolled into clinical studies without stratification based on underlying disease mechanisms, treatment effects that occur within specific subgroups may be difficult to detect. Consequently, therapeutic approaches that might benefit certain patient subsets may appear less effective when evaluated across heterogeneous study populations. This recognition has contributed to increasing interest in strategies that consider biological variability when designing clinical trials and therapeutic interventions. This heterogeneity has direct implications for prevention and clinical translation because uniform therapeutic approaches may dilute treatment effects, obscure responsive subgroups, and reduce the efficiency of prevention-oriented intervention strategies.

### 2.3 Limitations of current biomarkers

The evaluation of therapeutic interventions in T1D has also been influenced by limitations in the biomarkers commonly used to assess disease activity and treatment response. Clinical trials typically rely on measurements of stimulated C-peptide secretion to estimate endogenous insulin production and glycated hemoglobin levels to assess long-term glycemic control. These indicators provide valuable information about metabolic outcomes and remain important tools in clinical research. However, they primarily reflect downstream physiological consequences of β-cell loss rather than the earlier biological processes that drive autoimmune injury.

Changes in C-peptide levels often occur after substantial β-cell damage has already taken place, which means that alterations in this marker may not fully capture early effects of therapies aimed at modifying immune activity or cellular stress pathways (82). Similarly, glycemic outcomes are influenced by multiple behavioral and therapeutic factors, including insulin administration patterns, dietary intake, and physical activity (83–86). These influences can complicate interpretation of metabolic changes observed during therapeutic studies.

Another limitation arises from the absence of well-established biomarkers capable of identifying individuals who may respond most strongly to particular therapies. Without reliable indicators of immune activity, β-cell stress, or metabolic resilience, clinical studies often include participants with diverse biological profiles (87). This variability can make it more difficult to interpret treatment outcomes and may reduce the ability of studies to detect meaningful biological effects (88). The development of additional biomarkers capable of capturing early immunological or cellular changes could therefore improve the ability to evaluate therapeutic interventions and may facilitate more precise patient selection in future clinical trials. For public health translation, biomarker systems should evolve from static indicators of metabolic status toward dynamic tools that support risk prediction, intervention timing, treatment monitoring, and equitable deployment of disease-modifying therapies.

### 2.4 Translational gaps between animal models and human disease

Experimental models have contributed substantially to the study of autoimmune diabetes and have provided valuable opportunities to investigate mechanisms of immune-mediated β-cell injury (89). Animal models and controlled laboratory systems allow researchers to explore cellular interactions within pancreatic tissue, evaluate potential therapeutic interventions, and examine disease progression in environments where experimental variables can be carefully controlled (90–92). These systems have generated many important insights into immune pathways and cellular processes associated with T1D.

However, translating findings from experimental models into successful clinical therapies has proven difficult in some cases. Differences in immune system architecture between experimental models and humans can influence how autoimmune responses develop and respond to therapeutic intervention. Variations in antigen presentation pathways, cytokine signaling networks, and immune regulatory mechanisms may affect the biological context in which candidate therapies operate. Genetic susceptibility patterns also differ, as human T1D involves complex interactions among numerous genetic loci that influence immune regulation and β-cell vulnerability.

Pancreatic β-cell biology also exhibits species-specific differences that may influence disease mechanisms and therapeutic responses. Human β-cells display distinct metabolic properties and stress-response pathways compared with those observed in many experimental systems. In addition, the time course of disease progression in experimental models often differs from the variability observed in human populations, where autoimmune activity may develop over extended periods. These differences do not diminish the value of experimental models but highlight the importance of interpreting preclinical findings within the broader context of human disease biology. Continued development of experimental systems that more closely reflect human immune regulation and pancreatic cell function may help improve the translation of experimental discoveries into clinical therapies. Closing this translational gap is essential for population-level impact because interventions that appear effective in experimental systems must ultimately demonstrate safety, durability, feasibility, and generalizability across diverse human populations.

## 3 Redefining type 1 diabetes biology through integrated data intelligence

Advances in high-resolution molecular profiling, large-scale clinical datasets, and computational analytics are reshaping the conceptual framework through which T1D is understood. Historically, the disease was largely interpreted as a relatively uniform autoimmune condition defined by immune-mediated destruction of pancreatic β-cells and progressive loss of endogenous insulin secretion. Increasing evidence now indicates that T1D represents a spectrum of biologically related disease states shaped by diverse genetic architectures, immune regulatory programs, metabolic trajectories, and environmental influences. This evolving perspective has important implications for therapeutic development as it suggests that a single universal intervention is unlikely to achieve durable remission across all individuals. Instead, effective disease-modifying strategies will require precise characterization of the biological processes driving disease progression in individual patients and alignment of therapeutic approaches with these underlying mechanisms.

### 3.1 Heterogeneity as a central feature of disease biology

Recognition of biological heterogeneity represents a major shift in the modern understanding of T1D (73). Clinical observations have suggested substantial variability in disease trajectories among affected individuals, including differences in age at onset, rate of β-cell functional decline, and long-term metabolic outcomes (Table 2). Pediatric-onset disease often involves rapid immune-mediated β-cell destruction accompanied by accelerated loss of endogenous insulin secretion, whereas adult-onset autoimmune diabetes may progress more gradually with prolonged preservation of β-cell function. These clinical differences reflect deeper biological variability that cannot be explained by traditional diagnostic criteria alone. Genetic architecture contributes significantly to this variability, particularly through variation within human leukocyte antigen loci that regulates antigen presentation and adaptive immune activation. Additional non-HLA genetic variants influence immune regulatory pathways, inflammatory signaling networks, and β-cell resilience, producing diverse susceptibility profiles and disease trajectories across individuals.

**Table 2:** Major dimensions of biological heterogeneity in type 1 diabetes.

Dimension	Examples	Biological Impact	Therapeutic Implications	References
Age of onset	Pediatric vs. adult onset	Different immune activation intensity	Adjust immune-modulation strategies	(73, 75)
Genetic architecture	HLA-DR3/DR4, non-HLA loci	Alters antigen presentation	Precision immune therapies	(74, 113, 124)
Autoantibody patterns	GAD, IA-2, ZnT8	Predict progression rate	Stratified trial enrollment	(27, 75)
Immune phenotypes	T-cell dominant, interferon-high	Different inflammatory pathways	Targeted immunotherapies	(103, 123)
β-cell stress states	ER stress, oxidative stress	Increased immune recognition	β-cell protective therapies	(104, 131, 132)
Environmental factors	Microbiome, viral exposures	Immune priming	Preventive strategies	(35, 36, 110)

Immunological diversity further contributes to heterogeneity in T1D pathogenesis. Differences in autoreactive T-cell activation, regulatory T-cell function, B-cell–mediated antigen presentation, and cytokine signaling networks have been observed across patient populations (93–98). Some individuals exhibit immune signatures dominated by interferon-mediated inflammatory responses, whereas others demonstrate patterns characterized primarily by adaptive immune activation or innate inflammatory signaling (99–103). Variability in β-cell intrinsic biology introduces another dimension of disease diversity (104, 105). Under inflammatory or metabolic stress, β-cells undergo transcriptional reprogramming that alters antigen presentation, enhances inflammatory signaling, and increases susceptibility to immune-mediated cytotoxicity (41, 106, 107). The magnitude of these stress responses varies among individuals and may influence both the rate of β-cell decline and the potential for functional recovery (23, 108, 109). Environmental influences further shape disease trajectories through interactions with genetic susceptibility and immune regulation (36, 110, 111). Viral exposures, microbiome composition, early-life immune imprinting, and nutritional factors have all been implicated as modulators of autoimmune risk and disease progression (35). Together, these sources of biological variability demonstrate that T1D represents a heterogeneous disorder rather than a single uniform disease entity, underscoring the importance of mechanistic disease characterization in both research and therapeutic development.

### 3.2 Multi-omics integration and systems level mapping of disease networks

Technological advances enabling comprehensive measurement of biological systems across multiple molecular layers have transformed the study of complex diseases. In T1D, genomic investigations have identified numerous susceptibility loci influencing immune regulation and β-cell biology, while transcriptomic analyses have revealed gene expression programs associated with inflammatory signaling, interferon responses, and cellular stress pathways (112–115). Proteomic and metabolomic studies have further illuminated cytokine networks, metabolic signaling pathways, and biochemical processes that contribute to disease progression (106, 116). Although each of these data layers provides valuable insights into specific aspects of disease biology, no single modality captures the full complexity of the pathological processes that culminate in autoimmune β-cell destruction. Integration of these datasets within unified systems-level frameworks therefore represents a critical step toward understanding how diverse biological processes interact to produce the clinical manifestations of T1D (112, 114).

AI-driven analytical methods have the potential to enable simultaneous examination of multiple molecular and physiological variables, allowing identification of interconnected regulatory networks linking genetic susceptibility, immune activation, metabolic signaling, and β-cell stress responses (117, 118) (Table 3). Through such integrative analysis, investigators can map causal pathways connecting environmental triggers, immune activation, and endocrine pancreatic dysfunction (119). Genetic variants influencing antigen presentation may interact with viral or environmental exposures to initiate immune activation, which subsequently induces inflammatory cytokine production that imposes metabolic stress on β-cells (113, 120–122). Stressed β-cells may in turn increase antigen presentation or release inflammatory mediators that amplify immune recognition, creating feedback loops that accelerate autoimmune injury (61). Systems-level models derived from multi-omics integration therefore provide a framework for understanding how numerous biological processes interact dynamically during disease progression (114, 117, 118). By identifying regulatory nodes that exert disproportionate influence within these networks, such models may reveal therapeutic targets capable of modulating broader pathological processes rather than isolated molecular pathways.

**Table 3:** Multi-omics data types having implications in AI-driven T1D research.

Data Layer	Technologies	Biological insights	Clinical application	References
Genomics	GWAS, sequencing	Genetic susceptibility	Risk prediction	(113, 124)
Transcriptomics	RNA-seq, single-cell RNA-seq	Immune activation pathways	Endotype discovery	(114, 123)
Proteomics	Mass spectrometry	Cytokine networks	Biomarker discovery	(32, 106)
Metabolomics	LC-MS, NMR	Metabolic stress signals	Disease monitoring	(136, 317)
Microbiome	16S rRNA sequencing	Gut-immune interactions	Environmental risk modeling	(110, 317)
Digital biomarkers	CGM, wearable sensors	Real-time metabolic dynamics	Predictive disease modeling	(297, 319)

### 3.3 AI for disease endotype discovery

One of the most significant emerging applications of AI in T1D research involves the identification of mechanistically defined disease endotypes through analysis of high-dimensional biological datasets (114, 123–126). Traditional clinical classification of T1D relies primarily on diagnostic indicators such as hyperglycemia and circulating islet autoantibodies, features that confirm autoimmune diabetes but provide limited insight into the molecular processes responsible for disease progression in individual patients. ML approaches provide an alternative strategy by uncovering latent biological structure within multidimensional datasets without relying on predefined clinical categories (127, 128). Unsupervised analytical techniques including clustering algorithms, manifold learning methods, and network-based modeling can integrate genomic variation, immune phenotyping, transcriptomic profiles, metabolomic signatures, microbiome composition, and longitudinal metabolic data simultaneously (114, 118). By identifying recurring patterns across these interconnected data layers, ML models can group individuals according to shared biological characteristics, thereby revealing mechanistically coherent subgroups within the broader T1D population (129, 130) (Figure 1).

Integrative analyses have begun to identify several biological configurations underlying variability in disease progression. In some individuals, disease behavior appears dominated by strong adaptive immune activation characterized by expansion of autoreactive T lymphocytes, increased antigen presentation, and pro-inflammatory cytokine signaling. In others, molecular signatures suggest prominent β-cell stress and metabolic vulnerability, including evidence of endoplasmic reticulum stress, mitochondrial dysfunction, and oxidative injury that increase susceptibility to immune-mediated damage (131–135). Additional patterns involve activation of innate inflammatory pathways associated with host responses to microbial or environmental stimuli (110, 123, 124, 136, 137). These distinct biological configurations indicate that autoimmune β-cell destruction may arise through multiple interacting mechanisms rather than a single uniform pathway. Identification of such endotypes therefore has major implications for therapeutic development because interventions targeting specific biological pathways may demonstrate substantial benefit only within patient subgroups in which those pathways play a dominant role (Figure 1). For public health application, AI-derived endotypes should be evaluated not only for biological validity but also for interpretability, reproducibility across populations, and utility in guiding prevention-oriented or disease-modifying interventions. Such validation is essential to avoid models that perform well in narrow datasets but fail to generalize across age groups, ancestries, healthcare systems, and socioeconomic contexts.

### 3.4 Limitations of current datasets for AI development and validation

The expanding availability of molecular, clinical, and digital health data has created substantial opportunities for AI applications in T1D, although the evidentiary quality of available datasets remains uneven. Many datasets originate from relatively small cohorts, single-center studies, selected clinical trial populations, or individuals with consistent access to advanced diabetes technologies, limiting representation across age, ancestry, disease duration, treatment modality, socioeconomic context, geographic setting, and healthcare access. These sampling constraints can compromise external validity and contribute to reduced discrimination, poor calibration, or differential performance when models are applied beyond the populations used for development.

Methodological heterogeneity represents an additional limitation. Missing data, inconsistent phenotypic definitions, differences among CGM and insulin-delivery platforms, variable sampling intervals, batch effects in multi-omics datasets, and non-uniform preprocessing and endpoint definitions can introduce systematic variation that is unrelated to underlying disease biology. High-dimensional molecular datasets are particularly susceptible to overfitting when the number of measured features substantially exceeds the number of participants, which can yield unstable biomarker signatures and limited reproducibility across independent cohorts. Retrospective EHR datasets introduce further sources of bias through coding variability, informative missingness, treatment-selection effects, temporal changes in clinical practice, and incomplete capture of relevant biological or behavioral variables.

Longitudinal digital health datasets may also preferentially represent individuals with sustained engagement in connected technologies, thereby limiting inference to populations with lower device access, intermittent use, or reduced healthcare connectivity. Such selection effects are especially relevant for T1D, in which access to CGM, automated insulin delivery, and specialized clinical care varies substantially across populations and healthcare systems.

Improving the robustness of AI development will require multicenter and longitudinal datasets, standardized phenotyping and outcome definitions, harmonized preprocessing pipelines, transparent reporting of cohort composition, and independent external validation. Federated and privacy-preserving analytical frameworks may support cross-institutional model development while reducing the need for unrestricted transfer of patient-level data. Prospective evaluation across clinically relevant subgroups and independent care settings will be essential for determining whether AI models capture generalizable biological and clinical signals or predominantly reflect institution-, device-, or cohort-specific characteristics.

### 3.5 Methodological framework for AI/ML analysis and interpretation

AI/ML methods should be selected according to the analytical objective, outcome structure, dimensionality, and temporal characteristics of the underlying data. In supervised prediction, the target outcome is defined before model development, and model performance should be evaluated using metrics appropriate to the prediction task. Discrimination alone is insufficient for clinical prediction models; calibration, uncertainty, internal validation, and assessment of performance in populations distinct from the development sample are also required. Model complexity should be justified by demonstrable predictive value, as flexible ML algorithms remain susceptible to overfitting and optimistic performance estimates when sample size, event frequency, or validation procedures are inadequate (138–140).

Unsupervised analyses address a fundamentally different objective and should not be interpreted according to the same evidentiary framework as supervised prediction. Clustering and related exploratory approaches are used to characterize latent structure in datasets without requiring a predefined outcome, making them suitable for hypothesis generation in heterogeneous molecular or phenotypic data. In high-dimensional biomedical datasets, preprocessing, normalization, feature scaling, dimensionality, distance definitions, and analytical choices can materially influence the resulting structure. Computationally derived clusters therefore require assessment of stability, biological coherence, and replication in independent data before they can be interpreted as reproducible biological subgroups (141).

Multi-omics integration requires additional consideration of heterogeneity across molecular platforms. Integration may occur at the level of features, latent representations, similarity structures, or biological networks, and the appropriate strategy depends on whether the objective is dimensionality reduction, patient stratification, biomarker identification, or inference of relationships among biological components. Network-based approaches are particularly useful when the scientific question concerns coordinated molecular systems, as nodes and edges can encode interactions among genes, proteins, metabolites, or other biological entities. Network topology, data sparsity, modality-specific scaling, batch effects, and biological validation remain important determinants of interpretability, and inferred associations should not be described as causal regulatory relationships without independent experimental support (142).

Longitudinal data introduce analytical dependencies that are absent from conventional cross-sectional datasets. Repeated physiological measurements, EHR trajectories, and other time-indexed observations require validation strategies that preserve temporal ordering and prevent observations from the same individual or future time points from leaking into model development. Deep-learning architecture can accommodate non-linear relationships across variable-length time series, although increased representational capacity does not eliminate the requirements for independent validation, calibration, and evaluation under distribution shift. Accordingly, performance derived from retrospective time-series datasets should be interpreted as predictive performance within the evaluated setting unless prospective deployment demonstrates that predictions remain accurate and clinically useful in newly observed patients (143, 144).

Interpretability and explainability require a separate methodological distinction. *Post-hoc* explanation methods can quantify how model inputs contribute to a prediction, but an explanation should be evaluated for both fidelity to the underlying model and usefulness to its intended clinical user. Feature attribution does not establish biological causality, and a variable assigned high predictive importance should not automatically be interpreted as a mechanistic determinant or therapeutic target. This distinction is particularly important when AI/ML is applied to high-dimensional clinical or molecular data in which correlated predictors can produce multiple statistically adequate explanations of the same output (145, 146).

Generative models address a different analytical problem from prediction or classification. Architectures such as variational autoencoders, generative adversarial networks, transformers, diffusion models, and related frameworks can generate candidate molecular structures or synthetic representations by learning distributions within training data. Their output constitutes computational generation or prioritization and does not itself establish biological activity, target engagement, safety, or therapeutic efficacy. Candidate molecules produced by generative models therefore require sequential computational evaluation, experimental validation, pharmacological characterization, and ultimately clinical testing before claims of therapeutic benefit are warranted (147, 148).

A similar distinction applies to privacy-preserving distributed learning. Federated learning permits models to be trained across institutional datasets without pooling raw patient-level data, yet decentralization does not inherently guarantee privacy, generalizability, or clinical validity. Site-specific differences in data distributions, missingness, class imbalance, model aggregation, communication architecture, and privacy attacks can materially influence performance. Evaluation should therefore address intersite heterogeneity, held-out-site generalization, privacy safeguards, distributed optimization, and comparison with appropriate centralized or local baselines before translational claims are made (149, 150).

Analytical performance, external validation, and clinical utility therefore represent distinct stages of evidence and should be interpreted accordingly. Retrospective analyses establish computational associations or predictive performance within existing datasets, whereas external validation evaluates model transportability to independent populations or settings. Demonstration of clinical utility requires prospective assessment of whether model-informed decisions improve clinically meaningful outcomes relative to an appropriate comparator (144, 151, 152). Accordingly, throughout this review, retrospective findings are described in terms of identification, association, classification, or prediction; proposed applications are characterized as investigational; and statements of clinical benefit are reserved for prospectively evaluated interventions. The major AI/ML methodological frameworks relevant to T1D research, together with their analytical objectives, appropriate data structures, inferential scope, and key validation requirements, are summarized in Table 4.

**Table 4:** Analytical frameworks and methodological considerations for AI/ML applications in T1D research.

Analytical framework	Primary scientific question	Applicable data types	Inference	Methodological requirements	References
Supervised classification	Can a predefined categorical outcome be predicted?	Clinical variables, EHRs, biomarkers, omics	Probability or class assignment for a defined endpoint	Adequate events/sample size; internal validation; discrimination; calibration; external validation	(139, 152)
Supervised regression	Can a continuous outcome be estimated?	Physiological, clinical, molecular data	Estimated value or trajectory of a predefined quantitative outcome	Outcome specification; non-linear effects; regularization; prediction error; calibration	(152)
Unsupervised clustering	Does the dataset contain latent groups without predefined labels?	Phenotypic, immune, molecular, multi-omics data	Data-derived similarity structure	Normalization; distance metric; cluster stability; sensitivity analysis; independent replication	(235)
Dimensionality reduction	Can high-dimensional information be represented using fewer latent dimensions?	Transcriptomic, proteomic, metabolomic, single-cell data	Reduced representation of correlated features	Scaling; preservation of biological information; component stability; avoidance of overinterpretation	(130, 142)
Network/graph analysis	Which measured biological entities exhibit structured relationships?	Multi-omics, interaction, pathway data	Network topology, modules, candidate relationships	Edge definition; network stability; multiple testing; biological validation; distinction between association and causality	(114, 142)
Time-series ML	Can future states be estimated from temporally ordered observations?	CGM, wearable, EHR and treatment trajectories	Temporal prediction conditional on previous measurements	Temporal data splitting; leakage prevention; prediction horizon; calibration; handling irregular sampling	(143)
Deep learning	Can complex non-linear representations be learned from high-dimensional or sequential data?	Imaging, omics, EHR, physiological time series	Learned predictive representations	Adequate sample size; regularization; hyperparameter control; external validation; interpretability	(143)
Explainable AI	Which input features influence a model output and how can predictions be communicated?	Outputs of complex supervised models	Model-level or prediction-level explanation	Fidelity; stability; user relevance; distinction between feature importance and causal inference	(145)
Multi-omics integration	Which signals are shared or complementary across molecular layers?	Genomic, transcriptomic, proteomic, metabolomic and epigenomic data	Cross-modality latent factors, signatures, or relationships	Modality normalization; missing blocks; dimensional imbalance; batch effects; replication	(37)
Generative modeling	Can new candidate structures or representations be generated from learned distributions?	Molecular structures, sequence data, latent representations	Computational candidates or synthetic observations	Novelty; validity; diversity; training-set bias; experimental confirmation	(147, 148)
Federated learning	Can models be developed across institutions without centralizing raw data?	Distributed multicenter clinical or multimodal datasets	Distributed model estimation	Non-IID data; site heterogeneity; aggregation strategy; privacy leakage; held-out-site validation	(149, 150)
Clinical prediction model validation	Does a developed model retain performance in new patients and settings?	Independent temporal, geographic, or institutional cohorts	Transportability of predictive performance	Discrimination; calibration; case-mix assessment; model updating when required	(151, 152)

## 4 Therapeutic discovery and optimization in type 1 diabetes

Increasing understanding of the immunological and metabolic mechanisms underlying T1D has generated numerous therapeutic strategies aimed at preventing autoimmune β-cell destruction and restoring endogenous insulin secretion. Despite extensive clinical investigation, translation of mechanistic insights into durable therapeutic outcomes has remained limited. Immunomodulatory agents, antigen-specific tolerance approaches, β-cell protective therapies, regenerative interventions, and metabolic adjuncts have each demonstrated partial preservation of β-cell function or transient improvements in metabolic regulation, yet sustained remission and long-term insulin independence remain uncommon. These limitations largely reflect the multifactorial nature of T1D pathogenesis, which arises from interacting immune, cellular, and metabolic processes rather than a single dominant pathological pathway. Therapeutic development has therefore increasingly shifted toward strategies capable of influencing multiple biological systems involved in disease progression while maintaining physiological immune and metabolic homeostasis. A public health–oriented therapeutic framework should therefore consider not only biological efficacy but also treatment timing, safety, monitoring requirements, cost, accessibility, and feasibility of implementation across health systems.

### 4.1 Immune modulation and restoration of immune tolerance

Autoimmune destruction of pancreatic β-cells constitutes the defining pathogenic process of T1D and therefore represents a central target for disease-modifying therapy (153, 154). The immunological cascade responsible for β-cell injury involves coordinated activity among multiple immune cell populations, including cytotoxic CD8^+^ T lymphocytes, helper CD4^+^ T cells, antigen-presenting cells, B lymphocytes, and inflammatory cytokine networks that sustain chronic immune activation within pancreatic islets (100, 123, 155, 156). Inflammatory signaling within this microenvironment alters β-cell antigen presentation and promotes recruitment of additional effector immune cells capable of mediating cytotoxic injury (157–159). Therapeutic strategies directed at immune modulation seek to interrupt these pathogenic immune circuits while preserving essential host defense mechanisms (160–164). Achieving this balance remains challenging as excessive immune suppression compromises protective immunity, whereas incomplete regulation permits autoreactive pathways to continue damaging endocrine pancreatic tissue.

Early therapeutic efforts focused on pharmacological agents capable of attenuating immune activation through targeted interference with lymphocyte signaling pathways (162, 164–167). Monoclonal antibodies directed against CD3 have demonstrated the capacity to preserve residual β-cell function in some individuals when administered during early disease stages by altering T-cell signaling dynamics and promoting immune regulatory phenotypes that partially restrain autoreactive cytotoxic responses (79, 161, 168, 169). Therapeutic depletion of B lymphocytes through CD20-directed antibodies has likewise slowed the decline of endogenous insulin secretion in certain patients, highlighting the contribution of B cells to antigen presentation processes that amplify T-cell–mediated immune responses (170, 171). Although these interventions can transiently stabilize β-cell function, durable remission remains rare as autoreactive immune memory and inflammatory signaling networks frequently reestablish pathogenic activity following the initial therapeutic effect.

Therapeutic development has therefore shifted toward approaches designed to restore antigen-specific immune tolerance rather than broadly suppressing immune activation. Antigen-directed immunotherapies attempt to recalibrate immune recognition by presenting β-cell–derived antigens in contexts that favor regulatory signaling rather than inflammatory activation (172–176). Strategies under investigation include peptide-based immunotherapies, tolerogenic dendritic cell platforms engineered to promote regulatory immune environments, and adoptive cellular therapies involving expansion of regulatory T-cell populations capable of suppressing autoreactive immune activity within pancreatic tissues (172–176). Advances in cellular engineering and gene-editing technologies further expand possibilities for precise immune regulation by enabling development of engineered regulatory immune cells with enhanced suppressive capacity and antigen specificity (177–180). By reshaping immune recognition pathways governing tolerance and immune regulation, these approaches aim to establish a physiological state in which β-cell antigens are recognized as self rather than targeted by cytotoxic immune responses.

### 4.2 β-cell preservation and cellular protection

Preservation of residual β-cell function represents an important therapeutic objective during early stages of T1D as even modest endogenous insulin secretion is associated with improved metabolic stability and reduced risk of severe complications (181–184). Surviving β-cells in individuals with newly diagnosed disease remain exposed to inflammatory cytokines, metabolic stress, and immune-mediated cytotoxicity that progressively impair insulin secretion and cellular viability (185–188). Cytokine-driven signaling pathways activate transcriptional programs that promote endoplasmic reticulum stress, mitochondrial dysfunction, oxidative injury, and altered antigen presentation within pancreatic β-cells, processes that collectively compromise cellular function and increase susceptibility to apoptosis (115, 189, 190). Therapeutic strategies aimed at β-cell preservation therefore focus on reducing cellular stress and enhancing intrinsic mechanisms that enable β-cells to maintain function under inflammatory conditions (191–193).

Pharmacological agents capable of modulating endoplasmic reticulum stress responses, enhancing mitochondrial bioenergetics, or reducing oxidative damage have demonstrated protective effects in experimental systems by improving β-cell resilience to inflammatory signaling (80, 194–196). Metabolic interventions that reduce glucotoxicity and lipotoxicity may further decrease physiological workload on surviving β-cells, allowing endocrine cells to sustain insulin secretion under conditions of reduced metabolic demand (197–199). Advances in molecular profiling are also identifying regulatory pathways that govern β-cell survival and adaptive responses to inflammatory injury (200, 201). These studies provide opportunities to develop targeted therapies capable of stabilizing endocrine cell function during early phases of disease progression. Such strategies may prolong endogenous insulin secretion and improve metabolic outcomes even in the presence of ongoing autoimmune activity (162, 163, 202).

### 4.3 β-cell replacement and regenerative therapies

For individuals with advanced disease characterized by extensive β-cell loss, restoration of functional insulin-producing cells represents a promising strategy for reestablishing endogenous insulin secretion (67, 203–206). Pancreatic islet transplantation has demonstrated that reintroduction of functional β-cells can substantially improve glycemic control and, in some cases, achieve insulin independence (207–210). However, widespread clinical implementation has been constrained by limited donor tissue availability and the need for long-term immunosuppression to prevent immune rejection and recurrent autoimmune destruction (211–215).

Advances in stem cell biology have created new opportunities to generate insulin-producing cells from pluripotent stem cell platforms capable of differentiating into β-like cells that closely resemble native endocrine cells in both structure and glucose-responsive insulin secretion (204, 216–218). Improvements in differentiation protocols have enhanced functional maturity and metabolic competence of these cells following transplantation (219–222). Early clinical investigations evaluating stem cell–derived β-cell replacement therapies have produced encouraging preliminary results, suggesting that scalable cellular sources may overcome limitations associated with donor islet scarcity (204, 205, 211). Long-term survival and functional integration of transplanted cells remain central challenges because implanted endocrine tissue must be protected from both alloimmune rejection and recurrence of autoimmune responses (211, 223–226).

Bioengineering strategies are being developed to address these challenges through encapsulation technologies that shield transplanted cells from immune recognition while allowing diffusion of oxygen, nutrients, and insulin (205, 225). Gene-editing approaches are also being explored to modify transplanted cells in ways that reduce immunogenic antigen presentation or enhance resistance to inflammatory signaling (223, 226). Complementary regenerative strategies aimed at stimulating endogenous β-cell proliferation or reprogramming alternative pancreatic cell populations into insulin-producing cells are under investigation, offering additional pathways for restoring functional endocrine tissue (218, 220, 227, 228).

### 4.4 Computational and AI-guided therapeutic discovery

The complexity of T1D pathogenesis presents significant challenges for traditional drug discovery approaches that rely on identification of single molecular targets. Computational frameworks incorporating AI and ML enable analysis of large-scale biological datasets to identify regulatory pathways associated with disease progression and therapeutic responsiveness (118, 229–231). Integration of genomic variation, immune phenotyping data, transcriptomic profiles, proteomic signaling networks, metabolomic signatures, and clinical outcomes allows computational models to reveal molecular interactions that may represent promising targets for therapeutic intervention (118, 232–234).

These analytical approaches also enable systematic evaluation of therapeutic response patterns across diverse patient populations (235, 236). Analysis of historical clinical trial datasets and longitudinal patient records can reveal biological features associated with favorable responses to specific interventions, thereby informing prioritization of future therapeutic strategies (87, 237). Computational frameworks further support drug repurposing efforts by identifying existing pharmacological agents capable of modulating pathways implicated in T1D pathogenesis (230). Generative modeling approaches extend these capabilities by exploring vast chemical design spaces and proposing candidate molecules predicted to interact with biological targets involved in immune regulation or β-cell biology (130, 238). In addition, computational simulations can evaluate potential interactions among multiple therapeutic agents, providing insight into how combinations of interventions may influence complex biological networks governing autoimmune activity and endocrine function (230). To align with public health priorities, AI-guided therapeutic discovery should incorporate clinically meaningful endpoints, demographic representativeness, real-world validation, and implementation constraints during model development.

### 4.5 Toward integrated combination therapeutics

Increasing evidence indicates that durable therapeutic success in T1D will require coordinated interventions capable of addressing multiple dimensions of disease biology. Suppression of autoimmune activity alone may slow β-cell destruction but cannot restore endocrine function once substantial cellular loss has occurred. Conversely, strategies focused solely on β-cell replacement may fail if the underlying autoimmune environment remains capable of damaging newly introduced endocrine cells. Therapeutic frameworks therefore increasingly emphasize integrated approaches that combine immune modulation, β-cell preservation or restoration, and stabilization of metabolic physiology (82, 163, 239, 240).

Systems-level analytical models provide valuable tools for identifying therapeutic combinations capable of influencing multiple regulatory pathways simultaneously (235, 241). By mapping interactions among immune signaling networks, β-cell stress responses, and metabolic regulatory systems, computational frameworks can identify combinations of interventions predicted to produce synergistic effects on disease progression (130, 237). These models may also inform the optimal sequencing and timing of therapeutic interventions, recognizing that different biological processes dominate at different stages of disease evolution (235, 241). Development of integrated therapeutic strategies capable of reestablishing immune tolerance, restoring functional β-cell mass, and maintaining stable metabolic regulation therefore represents a central objective in the pursuit of durable disease modification in T1D.

## 5 AI enabled clinical trial design and precision medicine frameworks

The translation of promising biological discoveries into effective therapies for T1D has historically been constrained by limitations in conventional clinical trial design. Traditional randomized controlled trials were developed primarily for evaluating interventions that exert relatively uniform effects across large patient populations.

AI and advanced computational analytics offer powerful tools for addressing these limitations. By integrating complex multidimensional datasets derived from clinical records, molecular profiling studies, and digital health technologies, AI can enable more precise patient stratification, improve prediction of therapeutic response, and guide the development of adaptive clinical trial frameworks. These innovations have the potential to substantially increase the efficiency of clinical research and accelerate the identification of therapies capable of producing durable disease remission. This orientation is relevant to digital public health because adaptive trial designs, digital monitoring, and real-world evidence can strengthen the pathway from therapeutic discovery to population-level implementation.

### 5.1 Precision patient stratification and trial enrichment

One of the most consequential applications of AI within clinical research for T1D lies in its ability to support precision patient selection in therapeutic trials (130). Traditional enrollment strategies for T1D studies have typically relied on broad clinical descriptors such as age at diagnosis, duration of disease, residual C-peptide secretion, and the presence of circulating islet autoantibodies (87). Although these criteria remain important for confirming autoimmune diabetes and defining general disease stage, they provide limited insight into the biological conditions that influence whether an individual is likely to respond to a specific therapeutic intervention.

Advances in AI and ML provide a methodological framework for addressing this limitation by enabling systematic analysis of complex clinical and molecular information generated during both research studies and routine clinical care (117, 232). Computational models can integrate multidimensional data sources, including genomic variation, immune phenotyping, circulating biomarker measurements, and longitudinal clinical observations, to identify patterns that distinguish groups of individuals who share physiological characteristics relevant to therapeutic responsiveness (118). Incorporating these analytical insights into trial design allows investigators to move beyond generalized eligibility criteria and instead select participants whose biological profiles more closely correspond to the mechanism of action of the investigational therapy (88). Aligning trial enrollment with patient-specific biological context increases the likelihood that therapeutic effects will be detectable within the study population (88). This approach may improve the interpretability of trial outcomes and reduce the probability that promising therapeutic strategies are overlooked during early stages of clinical evaluation (88).

The use of biologically informed stratification strategies also has important implications for the statistical efficiency and mechanistic insight derived from clinical trials (88). Enrichment of study cohorts with participants whose disease characteristics are relevant to the therapeutic mechanism can substantially reduce variability within the trial population, increasing statistical power and enabling meaningful treatment effects to be detected with smaller sample sizes than would be required in heterogeneous cohorts. In addition, stratified analysis of treatment responses across defined participant groups allows investigators to examine how therapeutic efficacy varies according to underlying biological conditions. Such analyses can reveal patterns of response that inform subsequent trial design and support the development of therapeutic strategies more precisely aligned with patient-specific disease characteristics. Through the integration of computational stratification with targeted enrollment and mechanistically informed outcome evaluation, precision patient selection represents an important methodological advancement capable of improving the efficiency and interpretability of therapeutic trials in T1D. For public health translation, stratification models should improve trial efficiency while preserving external validity, ensuring that resulting therapies are applicable to the broader T1D population rather than only narrowly selected research cohorts.

### 5.2 Adaptive and platform trial designs

The growing diversity of investigational therapies for T1D has exposed important limitations in conventional randomized clinical trial structures, which are typically designed around fixed protocols, predetermined treatment assignments, and static analytical frameworks. Traditional trial designs assume that therapeutic effects will emerge only after the completion of a study and that the underlying structure of the investigation should remain unchanged throughout its duration. While this approach has historically provided a robust method for evaluating pharmacological interventions, it is less well suited to rapidly evolving therapeutic landscapes in which multiple experimental strategies are being developed simultaneously. In the context of T1D, where new interventions targeting immune pathways, cellular replacement, and metabolic regulation continue to emerge, trial structures that allow greater responsiveness to accumulating evidence can substantially improve the efficiency with which therapeutic candidates are evaluated.

Adaptive clinical trial designs provide a framework for introducing controlled flexibility into the conduct of clinical research (Table 5) (242–244). Within these designs, predefined interim analyses allow specific elements of the trial such as participant allocation ratios, continuation of treatment arms, or sample size adjustments to be modified as evidence accumulates during the course of the study (245). These modifications occur within carefully constructed statistical boundaries that preserve the validity of the trial while allowing investigators to respond to emerging signals of efficacy or lack of benefit (246, 247). By permitting early identification of promising interventions and reducing exposure to treatments that show minimal activity, adaptive trials create a more efficient investigative environment in which scientific learning occurs continuously rather than only after the study has concluded.

**Table 5:** AI-enabled innovations in clinical trial design.

AI Application	Function	Benefit for T1D trials	References	Representative example (ClinicalTrials.gov NCT Number)
Patient stratification	Identify mechanistic endotypes	Higher response rates	(87, 88, 130)	NCT07461805
Predictive modeling	Forecast disease progression	Early intervention	(126, 127, 297)	NCT06280729
Adaptive randomization	Adjust treatment allocation	Faster learning	(242, 245)	Not currently identified.
Digital biomarkers	Continuous metabolic monitoring	Early detection of response	(279, 289, 297)	NCT07039617
Trial simulation	*In-silico* modeling	Reduced sample size	(257, 301, 303)	NCT05610111
Adaptive and platform trial designs	Modify allocation, treatment arms, or trial features based on accumulating evidence	Faster learning and more efficient evaluation of multiple interventions	(242, 245, 256)	NCT07216391 and NCT07683026

Platform trials extend this concept further by establishing a single, ongoing clinical trial infrastructure capable of evaluating multiple therapeutic approaches under a unified protocol (248–250). Rather than launching separate studies for each investigational therapy, a platform design allows multiple treatment arms to be assessed simultaneously using shared eligibility criteria, outcome definitions, and data collection systems (251–253). As new therapies become available, they can be incorporated into the platform without requiring the initiation of entirely new trials, while interventions that fail to demonstrate sufficient clinical benefit can be removed from the study structure (254, 255). This continuous evaluation framework allows investigators to compare multiple strategies within a consistent experimental environment, facilitating more rapid identification of therapeutic approaches that warrant further development.

The integration of AI-supported analytical methods further strengthens the potential of adaptive and platform trial structures by enabling continuous evaluation of evolving study outcomes (256). Computational models can assist investigators in identifying emerging treatment signals, monitoring safety trends, and informing decisions regarding protocol modifications within predefined statistical parameters (257). When combined with flexible trial architecture, these analytical capabilities create a dynamic research framework capable of learning from accumulating evidence while maintaining methodological rigor. Such approaches offer a powerful strategy for accelerating therapeutic evaluation in T1D, allowing promising interventions to be identified and advanced more efficiently than would be possible within traditional fixed trial designs.

### 5.3 Integration of digital health and real time physiological monitoring

The evaluation of therapeutic interventions in T1D increasingly requires analytical approaches capable of capturing the evolving physiological responses that occur during the course of a clinical trial. Traditional study designs have typically relied on predetermined primary endpoints assessed at specific follow-up intervals, such as preservation of C-peptide secretion or improvements in glycemic control measured over a defined period. While these endpoints remain essential for determining clinical efficacy, they often provide only a limited view of the biological processes unfolding during treatment. Disease activity, metabolic stability, and physiological adaptation to therapeutic intervention may change progressively over time, and meaningful signals regarding treatment performance can emerge long before the final outcome assessment is completed. Monitoring strategies that incorporate repeated evaluation of physiological indicators throughout the study period therefore allow investigators to observe how treatment effects develop dynamically rather than relying solely on isolated endpoint measurements.

Longitudinal monitoring frameworks enable investigators to characterize treatment responses with greater temporal resolution by examining trajectories of physiological change across the duration of the trial. Participants receiving an investigational therapy may demonstrate a wide range of response patterns, including early metabolic stabilization, gradual improvement over extended follow-up, or minimal deviation from baseline disease activity. Careful evaluation of these response trajectories provides insight into the stability and progression of therapeutic effects, helping investigators determine whether observed improvements represent transient fluctuations or sustained physiological changes associated with meaningful clinical benefit. Such analyses can also reveal patterns that indicate delayed therapeutic responses, which might otherwise be overlooked if evaluation were restricted to a limited number of predefined time points.

Continuous monitoring approaches further enhance the interpretability of clinical trials by providing a more detailed representation of participant-level variability in treatment response. Differences in disease progression, metabolic adaptation, and physiological resilience can influence how individuals respond to experimental interventions, producing heterogeneous outcome patterns within the study population. Evaluating these patterns over time allows investigators to better understand the distribution and consistency of treatment effects across participants, thereby clarifying the overall therapeutic profile of the intervention under investigation. This level of analytical detail contributes to a more nuanced interpretation of trial outcomes and supports more informed decisions regarding the advancement of investigational therapies into subsequent stages of clinical development.

### 5.4 Predictive modeling

Accurate evaluation of therapeutic interventions in T1D requires outcome measures capable of reflecting the complex physiological processes that govern glucose regulation and metabolic stability (197, 258, 259). Historically, clinical trials have relied on a limited set of biochemical indicators, including glycated hemoglobin levels, insulin requirements, and preservation of C-peptide secretion, to assess treatment efficacy (260–262). These measurements remain essential for determining whether an intervention influences endocrine pancreatic function or long-term glycemic control (79). However, such indicators provide only a partial representation of the physiological mechanisms that determine metabolic regulation in daily life. Glucose homeostasis is shaped by continuous interactions among insulin secretion, peripheral glucose uptake, hepatic glucose production, and behavioral factors such as dietary intake and physical activity (263–267). Outcome measures capable of capturing these multidimensional physiological processes therefore offer an opportunity to evaluate therapeutic benefit with greater biological relevance than traditional single-variable endpoints (268–271).

Recent advances in digital health technologies have enabled the development of biomarkers derived from high-resolution physiological measurements collected during routine metabolic activity (272–275). Continuous glucose monitoring systems generate detailed information regarding patterns of glycemic fluctuation, including the duration of time spent within target glucose ranges, the frequency and magnitude of hyperglycemic excursions, and the occurrence of hypoglycemic episodes (276–278). These indicators provide a more comprehensive representation of metabolic control than summary measures such as average glucose levels, as they capture the stability and variability of glucose regulation under everyday conditions (279–281). When incorporated into clinical research, digitally derived biomarkers can therefore offer insight into how therapeutic interventions influence the real-world dynamics of metabolic regulation rather than solely reflecting static biochemical averages (282).

Advanced outcome measures also allow investigators to evaluate therapeutic performance across multiple physiological dimensions simultaneously (283, 284). Indicators such as glycemic variability, glucose stability during nocturnal periods, and responsiveness to metabolic challenges provide complementary information regarding the functional impact of investigational therapies (264, 285). By incorporating such multidimensional metrics, clinical trials can assess not only whether an intervention improves overall glycemic control but also whether it enhances the resilience and stability of metabolic regulation under varying physiological conditions (281, 286, 287). These expanded outcome frameworks enable a more comprehensive evaluation of therapeutic benefit and help clarify how emerging interventions influence the broader physiological systems responsible for maintaining glucose homeostasis (288).

The integration of digital biomarkers into clinical trial methodology therefore represents an important step toward more precise evaluation of therapeutic outcomes in T1D (289, 290). By expanding the range of measurable physiological indicators beyond traditional biochemical endpoints, investigators can obtain a more detailed understanding of how investigational therapies influence metabolic function in everyday life (291, 292). Such outcome measures complement established clinical metrics while providing additional insight into the functional stability of glucose regulation (293–295). As digital monitoring technologies continue to advance, the incorporation of these high-resolution physiological indicators into clinical research frameworks will play an increasingly important role in defining therapeutic success and guiding the development of new interventions for T1D (296).

### 5.5 Emerging clinical applications of AI in type 1 diabetes

AI-enabled approaches are increasingly being evaluated as clinically actionable components of T1D management, particularly for insulin-dose optimization, individualized glucose forecasting, and prospective identification of glycemic risk. These applications translate longitudinal CGM, insulin-delivery, clinical, and behavioral data into patient-specific predictions or therapeutic recommendations that can complement conventional episodic assessment (297–299). AI-based clinical decision support has demonstrated particular potential for optimization of insulin therapy. In the multicenter ADVICE4U randomized trial, automated insulin-dose adjustment every three weeks produced glycemic outcomes that were non-inferior to titration performed by physicians at specialized diabetes centers (300). Complementary studies have also demonstrated clinically plausible automated recommendations for pump parameters and explainable ML-based estimation of individual total daily insulin requirements (229, 238).

More recent work has extended this paradigm through patient-specific physiological simulation and digital-twin–assisted therapeutic decision support. A randomized clinical trial of a digital twin-enhanced bolus decision-support system reported increased TIR and fewer hypoglycemic events compared with conventional carbohydrate-counting–based dosing (301). These findings support prospective evaluation of individualized simulation as an adjunct to prandial insulin selection. Short-term glucose forecasting represents another clinically relevant application of AI in T1D. Deep-learning architectures integrating preceding glucose trajectories with insulin and meal information have generated individualized forecasts over clinically relevant prediction horizons (302). Newer approaches incorporate predictive uncertainty and patient-specific adaptation to improve interpretation of anticipated glucose excursions (303, 304). Prediction of hypoglycemia may provide a more direct route from glucose forecasting to preventive clinical action (297). An externally validated ML model using CGM data from the REPLACE-BG, JDRF, and Tidepool cohorts predicted clinically significant hypoglycemia one week in advance (297). This finding suggests the potential use of AI to prioritize individuals requiring intensified remote review or anticipatory therapeutic adjustment.

AI approaches are also being developed to identify impending metabolic deterioration before progression to clinically overt DKA. Supervised ML models integrating CGM-derived glucose trajectories have been developed to identify elevated ketone concentrations during hyperglycemia in individuals with T1D, supporting earlier detection of metabolic deterioration preceding DKA (305). EHR-based predictive models have also been developed to stratify individuals with T1D according to subsequent DKA risk. These include models predicting DKA-related hospitalization in youth and large multicenter analyses identifying patients at increased risk of adverse glycemic outcomes (306).

AI is also being evaluated for earlier recognition and phenotypic classification of T1D across different stages of disease. A ML algorithm developed from Welsh primary-care EHRs and externally validated using English healthcare data identified children at increased probability of T1D before conventional diagnosis (127). At an alert threshold corresponding to 10% of primary-care contacts, 71.6% of children who subsequently developed T1D would have generated an alert within the preceding 90 days, with diagnosis anticipated by approximately 9 days on average (127). Data-driven stratification has also been applied to individuals with established islet autoimmunity. Outcome-guided clustering of TrialNet Pathway to Prevention data identified clinically distinct groups defined by demographic, genetic, metabolic, and autoantibody characteristics that differed substantially in progression risk and were subsequently evaluated in an independent DPT-1 cohort (235). In adults, ML-based classification may address diagnostic uncertainty when autoimmune diabetes is initially coded as type 2 diabetes (307). A model originally developed using national EHR data retained discriminatory performance when transferred to a regional health-information exchange, and retraining on local data further improved performance, supporting the feasibility of algorithm-assisted identification of probable adult-onset T1D for clinician review (308).

A distinct translational application involves increasingly autonomous AID systems that couple continuous glucose sensing with algorithmic insulin adjustment while reducing dependence on meal-related user input. In the CLOSE IT randomized controlled trial, adults using an open-source AID system without meal announcement achieved a TIR comparable to participants continuing hybrid closed-loop operation with meal announcements over 12 weeks (296). The multicenter FCL@Home randomized crossover study extended this concept to adolescents, young adults, and adults, demonstrating improved mean sensor glucose and TIR during fully closed-loop AID without mealtime bolusing compared with usual care (309). Prandial automation is also progressing beyond quantitative carbohydrate counting (310, 311). A randomized crossover pilot trial evaluated ultrarapid lispro delivery using qualitative meal-size estimation and fully closed-loop meal detection, while earlier controlled studies demonstrated the feasibility of automated control during unannounced meals and simplified meal estimation (312). Together, these studies indicate that algorithmic control is progressively shifting from reactive insulin adjustment toward context-responsive systems capable of managing meal-related glucose excursions with reduced manual intervention.

AI applications are additionally extending beyond acute glycemic management toward longitudinal assessment of diabetes-related complications. In adults with T1D, ML models trained on longitudinal CGM-derived features have been evaluated for prediction of incident diabetic retinopathy, with classifiers incorporating composite measures of hyperglycemic exposure and hypoglycemia risk achieving an AUC-ROC of approximately 0.92 in an exploratory cohort (313). This work is supported by longitudinal evidence showing that lower TIR and greater hyperglycemic exposure are associated with subsequent retinopathy risk, providing a physiological basis for extracting complication-related information from long-duration CGM records (314). These approaches illustrate a broader clinical trajectory in which AI may integrate continuously generated metabolic data with EHR-derived information to support earlier diagnosis, disease-stage classification, automated therapeutic adjustment, and complication-risk assessment. The most clinically informative applications will therefore be those that convert longitudinal data into temporally actionable outputs linked to defined decisions, including diagnostic evaluation, intensified surveillance, individualized insulin adjustment, or complication screening.

## 6 Future directions and roadmap toward a potential curative strategy for T1D

Despite substantial progress in elucidating the immunological, genetic, and metabolic mechanisms underlying T1D, the development of durable disease-modifying and potentially curative strategies remains an unresolved objective. Converging advances across multiple scientific domains are beginning to reshape the landscape of therapeutic development. Progress in immunology, stem cell biology, molecular systems profiling, AI-driven computational analysis, and innovative clinical trial methodologies are enabling more comprehensive investigation of the biological networks that govern disease progression. These developments support a transition from therapeutic paradigms focused primarily on metabolic compensation toward strategies that target the underlying mechanisms responsible for immune dysregulation and progressive β-cell dysfunction (315). Achieving durable disease modification will require coordinated integration of precision medicine approaches, advanced computational analytics, and large-scale collaborative research infrastructures (118). In parallel, appropriate ethical oversight and adaptive regulatory frameworks will be essential to ensure responsible clinical translation of emerging biological therapies. Together, these developments establish a strategic foundation for future research aimed at restoring stable endogenous metabolic regulation and long-term β-cell function in individuals with T1D. For alignment with public health priorities, future therapeutic roadmaps should consider not only biological plausibility but also prevention strategies, implementation feasibility, equity, health-system capacity, and long-term population outcomes.

### 6.1 Precision medicine approaches for patient-specific therapy

The clinical translation of disease-modifying and potentially curative therapies for T1D will require alignment of therapeutic interventions with the biological state of individual patients at specific stages of disease progression (316). Current treatment strategies largely rely on standardized insulin replacement and glycemic control, which stabilize metabolism but do not address the biological heterogeneity underlying autoimmune β-cell destruction or regenerative capacity. As therapies targeting immune regulation, β-cell restoration, and metabolic stabilization advance, their effectiveness will depend on identifying the appropriate patients and therapeutic windows for intervention (79, 161). Precision medicine frameworks therefore emphasize biologically informed therapeutic selection based on patient-specific immune, metabolic, and cellular states rather than uniform treatment protocols.

Implementation of this approach requires analytical systems capable of integrating heterogeneous clinical and physiological datasets (126, 317). Longitudinal clinical records, continuous glucose monitoring data, insulin dosing patterns, and other physiological measurements provide high-resolution insight into metabolic dynamics during disease progression and treatment (118, 318). When analyzed using advanced computational models, these data can identify patterns of metabolic stability, changes in insulin demand, and evolving responses to therapy (118, 126, 319). Such analyses may reveal temporal windows during which specific interventions such as immune modulation, β-cell regeneration, or cellular transplantation are most likely to succeed (320). Therapeutic decisions can therefore incorporate real-time physiological signals rather than relying solely on static clinical milestones (320).

Within this framework, therapeutic management becomes an adaptive process in which interventions are sequenced according to evolving disease biology. Immune tolerance strategies may be prioritized during periods of active autoimmune signaling, whereas regenerative or cell replacement therapies may be introduced once inflammatory activity is sufficiently controlled to permit stable β-cell engraftment (321). Metabolic interventions may be intensified during phases of glycemic instability to reduce physiological stress on pancreatic endocrine cells and support recovery of insulin secretory function (322, 323). Effective treatment may depend not only on the efficacy of individual interventions but also on their timing within the dynamic immunometabolic trajectory of the disease.

Realization of patient-specific therapeutic strategies will require healthcare systems capable of integrating continuous physiological monitoring with advanced computational analysis. Digital health platforms combining real-time metabolic data with predictive modeling may enable clinicians to assess disease dynamics and forecast therapeutic responses (118). As longitudinal clinical datasets expand, iterative machine learning models can refine predictions of disease progression and optimize treatment selection. Such adaptive analytical frameworks could support dynamic therapeutic planning based on patient-specific physiological signals, ultimately enabling precision interventions tailored to the evolving biological landscape of T1D. The public health challenge will be to translate patient-specific therapeutic selection into scalable care models that remain interpretable, affordable, and accessible across diverse clinical settings.

### 6.2 Global data integration and collaborative research infrastructure

Sustained progress toward disease-modifying and potentially curative interventions for T1D will depend not only on advances in biological discovery but also on the establishment of research infrastructures capable of coordinating scientific activity across institutions, disciplines, and healthcare systems worldwide. The pathophysiology of T1D encompasses interconnected processes spanning immune regulation, endocrine pancreatic biology, metabolism, and therapeutic bioengineering, fields that have historically evolved within distinct scientific communities. As therapeutic strategies become increasingly sophisticated, the evaluation and refinement of these approaches require collaborative environments in which investigators from diverse disciplines can contribute complementary expertise. International research infrastructures provide a mechanism for aligning experimental resources, clinical experience, and methodological innovation, allowing emerging therapeutic concepts to be examined across multiple research settings and patient populations. Such coordination reduces the fragmentation that often arises when scientific efforts are conducted independently and enables investigators to pursue integrated research agendas capable of addressing the multifaceted biological challenges associated with durable restoration of β-cell function.

Effective collaboration at this scale requires deliberate harmonization of research methodologies so that observations generated across independent programs can be interpreted within a common scientific framework. Differences in clinical characterization strategies, experimental procedures, and outcome definitions have historically limited the ability to synthesize findings across studies, slowing the accumulation of reliable evidence regarding disease mechanisms and therapeutic performance. When research groups employ inconsistent phenotyping approaches or divergent clinical endpoints, results become difficult to reconcile, preventing individual studies from contributing to a coherent body of knowledge. Coordinated efforts to standardize clinical assessment protocols, experimental methodologies, and reporting practices therefore represent an essential component of collaborative research infrastructure. Through the establishment of shared methodological standards, investigators working in different regions and institutions can generate results that are directly comparable, enabling discoveries to accumulate cumulatively rather than remaining confined to isolated research contexts.

Large-scale collaborative networks also provide the structural capacity necessary to evaluate emerging therapeutic strategies within diverse clinical environments. T1D affects individuals across a wide range of genetic backgrounds, environmental exposures, healthcare systems, and socioeconomic conditions, factors that may influence disease progression as well as the effectiveness and accessibility of therapeutic interventions. Research programs that extend across multiple geographic regions allow investigators to examine how candidate therapies perform under varied clinical circumstances, providing insights into their robustness and generalizability. Multinational collaboration also facilitates recruitment into clinical investigations at a scale that would be difficult for individual centers to achieve independently, thereby accelerating the pace at which new therapeutic approaches can be rigorously evaluated. By enabling coordinated clinical investigation across distributed healthcare systems, collaborative networks enhance the capacity of the scientific community to generate reliable evidence regarding therapeutic safety, durability, and scalability.

The continued development of global research ecosystems will therefore play a central role in translating emerging scientific insights into practical therapeutic advances. Collaborative infrastructures that encourage open scientific communication, shared technological development, and coordinated investigation create environments in which discoveries can be rapidly examined across independent research settings and refined through collective expertise. Within such systems, advances originating in basic science laboratories can be efficiently connected to translational experimentation and clinical evaluation, allowing promising therapeutic concepts to move more rapidly from early-stage discovery to clinical implementation. Through sustained cooperation among investigators, clinicians, engineers, and other scientific stakeholders, internationally coordinated research infrastructures provide the organizational foundation required to accelerate progress toward durable therapeutic strategies capable of transforming the long-term management of T1D.

### 6.3 Ethical, regulatory, and societal considerations

The development of emerging therapeutic strategies for T1D introduces complex ethical and regulatory questions that must be addressed in parallel with scientific and clinical progress. Many investigational approaches now extend beyond conventional pharmacological treatments and involve cellular engineering, gene-modified stem cell–derived β-cells, bioengineered implantation systems, or other biologically active therapeutic platforms. These interventions differ fundamentally from traditional medications as they may integrate permanently within the body, interact dynamically with physiological systems, and produce biological effects that persist for extended periods following administration (324). Regulatory evaluation of such therapies therefore requires frameworks capable of assessing not only short-term safety and efficacy but also long-term biological stability, potential off-target effects, and mechanisms for monitoring therapeutic performance after clinical deployment. The evolving nature of these technologies necessitates regulatory pathways that combine rigorous safety oversight with sufficient flexibility to enable responsible translation of innovative therapeutic concepts that may substantially alter the long-term clinical trajectory of autoimmune disease.

Ethical considerations are particularly important as therapeutic research increasingly explores interventions applied at earlier stages of disease development. In some contexts, individuals may be considered for experimental therapies before significant clinical deterioration has occurred or during phases in which symptoms remain manageable through existing treatments. Under these circumstances, the ethical balance between potential benefit and possible risk becomes especially complex, as participants may be asked to consider interventions whose long-term outcomes remain uncertain. Transparent communication regarding potential risks, realistic therapeutic expectations, and the limits of current knowledge therefore becomes essential to maintaining patient autonomy and informed decision-making. These considerations are especially significant in pediatric populations, where guardians often participate in medical decision processes on behalf of children who may ultimately live with the long-term consequences of experimental therapies introduced early in life.

The societal implications of emerging therapies also warrant careful attention as the therapeutic landscape of T1D evolves. Advanced biological interventions frequently involve sophisticated manufacturing processes, specialized clinical expertise, and complex monitoring requirements, factors that may influence how rapidly and broadly such treatments can be implemented within healthcare systems. Ensuring that scientific progress translates into meaningful health improvements across diverse populations requires early consideration of how novel therapies can be incorporated into healthcare delivery structures in ways that remain sustainable and equitable. Without deliberate planning, transformative therapeutic innovations risk becoming accessible only within limited clinical settings, potentially widening disparities in health outcomes between regions with differing healthcare resources.

Addressing these ethical and societal dimensions will require sustained collaboration among scientists, clinicians, regulatory authorities, ethicists, and individuals living with T1D. Patient communities play an especially important role in shaping responsible therapeutic development because they provide critical perspectives regarding acceptable levels of risk, treatment priorities, and quality-of-life considerations associated with long-term disease management. Engagement between researchers and patient communities can therefore guide clinical trial design, improve transparency in communication regarding emerging therapies, and help ensure that therapeutic innovation remains aligned with the needs and values of those most directly affected by the disease. Through thoughtful integration of ethical governance, regulatory oversight, and patient engagement, the advancement of new therapeutic strategies can proceed in a manner that maintains public trust while supporting responsible scientific progress.

Regulatory evaluation of AI-enabled clinical tools requires additional considerations beyond those applied to conventional medical interventions because model performance may depend on the intended clinical use, characteristics of the development population, data acquisition environment, and subsequent software modifications. For AI-enabled software functioning as a medical device or as part of a medical device, regulatory assessment should establish a clearly defined intended use and evaluate analytical and clinical performance using data representative of the target population and deployment setting. Lifecycle oversight is particularly important because changes in patient populations, clinical workflows, device inputs, or data distributions may result in performance drift after deployment. Accordingly, post-market monitoring should assess whether safety and effectiveness are maintained across relevant patient subgroups and clinical environments. For AI-enabled devices, anticipated software or model updates should be predefined, with clear procedures for their validation, implementation, and regulatory oversight. Clinically consequential outputs should also remain subject to appropriate human oversight, particularly when model recommendations influence diagnosis, treatment selection, or therapeutic adjustment. Together, prospective clinical validation, transparent definition of intended use, controlled software modification, and longitudinal performance surveillance are central to responsible regulatory translation of AI-enabled tools in T1D.

#### 6.3.1 Data privacy, ethical governance, and algorithmic bias in AI-enabled T1D care

The expanding use of AI in T1D research and clinical care introduces distinct ethical and governance considerations related to the acquisition, integration, and secondary use of high-dimensional patient data. AI models may incorporate CGM data, insulin dosing histories, EHR-derived variables, genomic and multi-omics profiles, wearable-device measurements, and other longitudinal physiological information, creating substantial requirements for data privacy, security, interoperability, and responsible data stewardship. Robust de-identification procedures, secure data storage and transfer, transparent consent frameworks, clearly defined data-access policies, and appropriate governance of secondary data use are therefore essential for responsible model development and clinical deployment.

Algorithmic bias represents a major translational concern when model development datasets do not adequately reflect the demographic, clinical, technological, and socioeconomic diversity of the broader T1D population. Training cohorts derived predominantly from specialized centers or individuals with consistent access to advanced diabetes technologies may underrepresent variation in age, ancestry, geographic setting, disease stage, healthcare access, and socioeconomic context. Such imbalances can reduce model generalizability and generate differential performance across patient subgroups, with potential consequences for risk prediction, therapeutic stratification, and clinical decision support.

Rigorous evaluation should therefore extend beyond aggregate measures of predictive performance and include subgroup-specific discrimination and calibration, external validation across independent healthcare systems, assessment of performance across device platforms and clinical settings, and prospective monitoring for model drift. Transparent reporting of cohort composition, missing-data patterns, model limitations, and sources of uncertainty is also necessary to support reproducibility and clinically meaningful interpretation.

Clinical implementation additionally requires clear delineation of human oversight and accountability. AI-generated recommendations should function as decision-support tools within established clinical workflows, with mechanisms for reviewing uncertain, discordant, or clinically implausible outputs and for communicating model limitations to clinicians and patients. Integration of privacy protection, fairness assessment, interpretability, reproducibility, and patient-centered governance throughout the AI lifecycle will be critical for ensuring that AI-enabled approaches improve T1D care while minimizing the risk of amplifying existing disparities.

## 7 Conclusions

T1D remains one of the most complex autoimmune diseases confronting modern medicine. Despite major advances in insulin therapy, glucose monitoring technologies, and automated insulin delivery systems, current treatments continue to address the metabolic consequences of the disease rather than its underlying immunopathology. Decades of clinical investigation have generated substantial knowledge regarding the genetic, immunological, and cellular mechanisms that drive autoimmune destruction of pancreatic β-cells. However, translation of these insights into durable therapeutic strategies has been limited by the multifactorial nature of disease pathogenesis, substantial heterogeneity among patients, insufficient mechanistic biomarkers, and limitations in traditional therapeutic and clinical trial paradigms.

Recent developments in systems biology, multi-omics profiling, and artificial intelligence are beginning to reshape the conceptual framework through which T1D is understood. Integrative computational approaches now allow researchers to examine the disease as a dynamic network of interacting genetic, immune, cellular, and metabolic processes rather than a single pathway disorder. This systems perspective has revealed substantial biological diversity among individuals with T1D and has highlighted the importance of identifying mechanistically defined disease endotypes that may respond differently to therapeutic interventions.

Advances in immunotherapy, stem cell derived β-cell replacement, regenerative medicine, and metabolic modulation provide promising avenues for restoring endogenous insulin production and reestablishing immune tolerance. When combined with precision patient stratification and adaptive clinical trial methodologies supported by artificial intelligence, these emerging strategies may enable more efficient development of targeted therapeutic combinations. In parallel, the establishment of large scale collaborative data infrastructures and continuously learning health systems will be essential for integrating clinical experience with emerging molecular insights.

In summary, scalable disease modification and potential curative strategies for T1D will likely require coordinated approaches that address immune dysregulation, β-cell loss, and metabolic instability together. This therapeutic gap has substantial public health significance because T1D is lifelong, complications accumulate over time, and effective interventions must be feasible beyond specialized research settings. AI-enabled systems biology, multi-omics integration, and digital health data can refine disease classification, identify actionable biomarkers, guide therapeutic selection, and support adaptive clinical development. To achieve public health impact, these approaches must be interpretable, clinically actionable, externally validated, and tested across populations that reflect the diversity of individuals living with or at risk for T1D. A digital public health perspective can strengthen this pathway by linking molecular discovery with clinical decision support, real-world evidence, implementation science, and equity-focused health-system planning. Together, interdisciplinary research, computational innovation, and global clinical collaboration may transform the therapeutic landscape of T1D. Future efforts should prioritize prevention-oriented implementation, equitable access, real-world validation, and measurable improvements in population-level outcomes.

Future research should prioritize the development and external validation of AI models using multicenter, longitudinal datasets that capture clinically relevant variation in age, ancestry, disease stage, treatment modality, socioeconomic context, and access to diabetes technologies. Prospective studies should determine whether AI-enabled stratification and clinical decision support improve patient-centered and therapeutic outcomes beyond conventional approaches, while integration of multi-omics biomarkers with longitudinal physiological data should be used to define reproducible disease endotypes and treatment-response signatures.

Adaptive and biomarker-enriched clinical trials should evaluate mechanistically informed combinations of immune modulation, β-cell preservation or replacement, and metabolic support across defined stages of T1D. Clinical translation will additionally require transparent model reporting, independent validation, human oversight, privacy protection, systematic assessment of algorithmic bias, regulatory alignment, evaluation of cost-effectiveness, and equitable implementation across healthcare settings. Collectively, these priorities can support the transition of AI from a predominantly analytical framework to a clinically validated component of precision prevention and disease-modifying therapy in T1D.
